# Clinical Practice Patterns in Tic Disorders Among Movement Disorder Society Members

**DOI:** 10.5334/tohm.656

**Published:** 2021-10-28

**Authors:** Christos Ganos, Harini Sarva, Lille Kurvits, Donald L. Gilbert, Andreas Hartmann, Yulia Worbe, Pablo Mir, Kirsten R. Müller-Vahl, Alexander Münchau, David Shprecher, Harvey S. Singer, Wissam Deeb, Michael S. Okun, Irene A. Malaty, Mark Hallett, Marina AJ Tijssen, Tamara Pringsheim, Davide Martino, on behalf of the Tic Disorders and Tourette Syndrome Study Group of the International Parkinson and Movement Disorder Society

**Affiliations:** 1 Department of Neurology, Charité University Medicine, Berlin, Germany; 2 Parkinson’s Disease & Movement Disorders Institute, Weill Cornell Medicine New York, United States; 3 Division of Neurology, Department of Pediatrics, Cincinnati Children’s Hospital Medical Center, University of Cincinnati College of Medicine, Cincinnati, OH, US; 4 Department of Neurology, APHP, Paris, Île-de-France, 75013, France; 5 Unidad de Trastornos del Movimiento, Servicio de Neurología y Neurofisiología Clínica, Instituto de Biomedicina de Sevilla, Hospital Universitario Virgen del Rocío/CSIC/Universidad de Sevilla, Seville, Spain; 6 Centro de Investigación Biomédica en Red sobre Enfermedades Neurodegenerativas (CIBERNED), Madrid, Spain; 7 Departamento de Medicina, Facultad de Medicina, Universidad de Sevilla, Seville, Spain; 8 Clinic of Psychiatry, Socialpsychiatry and Psychotherapy, Hannover Medical School, Hannover, Germany; 9 Institute of Systems Motor Science, University of Lübeck, Lübeck, Germany; 10 Banner Sun Health Research Institute, Sun City, AZ, United States; 11 Johns Hopkins University School of Medicine, Baltimore, Maryland; Kennedy Krieger Institute, Baltimore, Maryland; 12 UMass Memorial Medical Center and UMass Medical School, US; 13 Norman Fixel Institute for Neurological Diseases, Department of Neurology, University of Florida Health, Florida, United States; 14 Human Motor Control Section, Medical Neurology Branch, National Institute of Neurological Disorders and Stroke, National Institutes of Health, Bethesda, Maryland, US; 15 UMCG Expertise Centre Movement Disorders, Department of Neurology. University of Groningen, University Medical Center Groningen, Groningen, The Netherlands; 16 Department of Clinical Neurosciences, University of Calgary, Calgary, Canada

**Keywords:** Tic disorders, Tourette disorder, Survey, Tic Disorders and Tourette Syndrome Study Group, Movement Disorder Society

## Abstract

**Background:**

Tic disorders belong to the broad spectrum of pediatric and adult movement disorders. The wide variability in clinical presentations, applied assessment tools, and treatments are poorly understood.

**Objectives:**

To map practices and knowledge base of movement disorder clinicians concerning clinical features, pathophysiology, and treatment approaches in tic disorders.

**Methods:**

A 33-item survey was developed by the Tic Disorders and Tourette syndrome Study Group members of the Movement Disorder Society. The survey was distributed to the complete society membership and included responses from 346 members, 314 of whom reported treating tic disorders.

**Results:**

Approximately one third of survey respondents (35%) frequently evaluated patients with tics. The data revealed widespread use of existing guidelines (about 70%) and screening for comorbid disorders (>90%). The most common investigations used to rule out secondary causes of tics were imaging (92%), laboratory tests (66%) and neurophysiology (38%). Functional tics were the second most common tic etiology following primary tics. Only 27% of respondents reported confidence in knowledge about tic pathogenesis. Top rated interventions to treat tics were psychoeducation, cognitive behavioral intervention for tics (CBIT) and treatment for neuropsychiatric comorbidities. Antipsychotics were ranked as the most effective pharmacologic tic intervention.

**Conclusions:**

The majority of movement disorders specialists do not frequently encounter tics. There was sparse knowledge about tic pathophysiology. Psychoeducation, CBIT, the treatment of neuropsychiatric comorbidities and use of antipsychotics emerged as the most common interventions to treat tics. These results provide insight into what will be needed to improve the diagnosis and treatment of tic disorders.

## Introduction

Tics belong to the spectrum of hyperkinetic movement disorders and are defined as brief, sudden, and repetitive movements or sounds that resemble voluntary actions [1]. More complex repetitive behaviors, such as echo-, pali- and coprophenomena, also fall under the tic rubric. Tics are particularly prevalent in children and adolescents but also occur in adults [2]. The majority of patients with tics receive a diagnosis of primary tic disorder, including Tourette syndrome (TS; in DSM-5 labeled as Tourette Disorder [3]), but tics may also occur secondarily in association with other neurodevelopmental, neurodegenerative, immune-mediated, and toxic etiologies [1]. Notably, the presence of tics in primary tic disorders is often only one feature of a range of neuropsychiatric symptoms, such as attention deficit hyperactivity disorder (ADHD) and obsessive-compulsive disorder (OCD), repetitive self-injurious behaviors, depression, and anxiety. Given the large diversity of clinical profiles related to tic disorders, broad clinical expertise is necessary to properly assess and treat both the movement disorder of tics and the co-occurring neuropsychiatric conditions.

Several different guidelines have been developed in Europe, Canada, and the United States, to assist clinical practitioners of all fields, including neurology, pediatrics, child and adolescent psychiatry, adult psychiatry, and neuropsychiatry, in their therapeutic decisions [4,5,6]. However, very little is known about implementing these evidence-based guidelines in different parts of the world. Indeed, clinical experience shows considerable variability in treatment approaches and the overall perception of tics and their associations, in the knowledge of mechanisms related to their etiology and pathophysiology, and in the application of different assessment methods and tools. In addition, a survey on the availability of healthcare services for people with TS confirmed significant disparities among different geographic locations [7]. Within the field of neurological movement disorders, this is particularly relevant, as the steady expansion of treatment options, including new pharmacotherapeutic agents, neuromodulation techniques, alongside an increasingly mobile population, requires harmonizing practices in all these domains at a global level.

Given the paucity of existing data and with the help of the International Parkinson’s Disease and Movement Disorders Society (MDS), we sought to examine unifying themes by surveying movement disorders clinicians on their approaches to diagnosis and treatment of patients with tic disorders and TS. We also explored issues related to the general perception of tics, including their pathophysiology and factors influencing their overall prognosis. Our goal was to map similarities and differences on these key issues to form a basis and detect areas where consensus is lacking in order to work towards a standardized model of global care for people with tic disorders.

## Methods

Prior to the development of the study survey, members of the MDS, who formed the Tic Disorders and Tourette Syndrome Study Group, discussed knowledge gaps at the MDS 2019 annual congress in Nice (France). The 2020 survey was created by the presiding officers of the study group and subsequently sent to the remaining active members for revision. A total of thirteen members of the study group reviewed and modified the questionnaire. The survey focused on the following: demographics of the study participants, including the type of practice, number of patients with tic disorders seen per month and country of practice; features of tics and comorbid conditions; testing and scales used to diagnose and monitor symptoms; views on pathophysiology and treatment practices. The resulting 33-item survey (Supplementary Material 1) was sent to all MDS members via the society’s secretariat. Only practicing neurologists with direct experience in evaluating patients with tics and TS were asked to participate. The survey opened on June 3, 2020, and closed on July 22, 2020. Informed consent was not required since no personal identifying information was collected. Study data were collected and managed using REDCap electronic data capture tools [8,9]. Survey responses, including demographic and clinical practice characteristics, were summarized using descriptive statistics. We obtained different non-response rates across survey items; we report the absolute number of responses per item in tables and figures. We only report survey responses with more than a single data entry. The study protocol was approved by Charité University Medicine, Berlin (EA4/114/21).

## Results

### Demographic characteristics of survey respondents

Overall, there were 346 responses from MDS members in 72 countries (list of countries in Supplementary Table 1). Of the 346 respondents, 32 reported not having any patients with tic disorders, leaving a total of 314 respondents who saw patients with tic disorders. A breakdown of responder demographics can be seen in ***Table 1***.

**Table 1:** Demographics of survey respondents. N refers to number of respondents.


DEMOGRAPHIC VARIABLES	N	%

Age		

25–35	106	31

36–45	116	33

46–55	68	20

56–65	39	11

≥66	17	5

Years in practice		

≤5 yr	101	29

6–10 yr	69	20

11–15 yr	60	17

16–20 yr	41	12

≥21 yr	74	22

Type of practice		

Academic clinician in a hospital	249	72

Academic clinician in a community service	61	18

Non-academic clinician in a hospital	20	6

Non-academic clinician in a community service	15	4

Number of patients seen		

No patients	32	9

1–10 per year	193	56

1–10 per month	84	24

>10 per month	37	11

Patient profile		

Exclusively or mostly adults	125	43

Adults and pediatric	110	37

Exclusively or mostly pediatric	59	20

Continent (N = participants)		

Europe	129	38

The Americas	115	33

Asia	72	21

Africa	25	7

Oceania	2	1


### Assessment of tics in primary tic disorders and their associated comorbid conditions

First, we inquired about using standardized scales for the overall evaluation of tics, including tic severity in clinical practice. Approximately 40% of respondents did not use any scale for clinical evaluation of tics, and 36% used scales only in about a quarter of their patients. Only about 10% of respondents used validated scales in greater than 75% of their patients for tic assessment (a complete breakdown of responses is provided in Supplementary Figure 1). Among the movement disorders clinicians who did use clinical rating scales for their practice, the most applied scales included the Yale Global Tic Severity Scale (74%), followed by the Tourette Syndrome Global Clinical Impression Scale (42%) and the Modified Rush Video Protocol (12%). A complete list of scales and related frequency of use are in Supplementary Table 2.

Beyond the assessment of tics, we also determined whether movement disorders clinicians systematically document complex tic-related behaviors such as echo- or coprophenomena. More than 70% of respondents confirmed this practice. In addition, assessment of self-injurious behaviors, non-obscene inappropriate behaviors, and paliphenomena were also part of the clinical evaluation of patients in 60%, 54%, and 53%, respectively (Supplementary Figure 2). Regarding comorbid conditions, about 91% of respondents reported screening for the presence of OCD, ADHD and anxiety disorders, as well as depression using a clinical interview, but less than a third employed clinical scales to inform their clinical evaluation (see Supplementary Figure 3 and Supplementary Table 2 for a complete list of scales and related frequency of use).

### Clinical investigations and diagnostic spectrum

When secondary etiologies are suspected, 91.6% of survey respondents reported using neuroimaging (MRI or CT), 66% performed laboratory assessments, such as acanthocytes and ceruloplasmin, and 45% investigated additionally urinary copper levels. Streptococcal serology was routinely requested by 31% of survey respondents. In addition, 38% reported routinely applying neurophysiological investigations (e.g., EEG, EMG-EEG; see Supplementary Figure 4).

**Figure 1:**
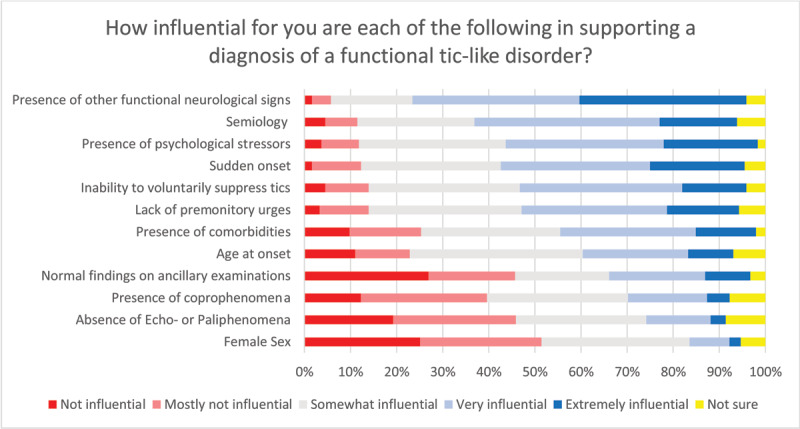
Factors supporting a diagnosis of functional tic disorder. Total number of respondents: n = 245.

Beyond primary tics, the most common differential etiology of a tic disorder was reported to be a functional neurological disorder (71%), followed by neurodegenerative disorders (e.g., Huntington’s disease, Chorea-Acanthocytosis, 65%) and medication-induced syndromes (50%) (Supplementary Figure 5). Twenty percent of survey respondents reported diagnosing a functional tic disorder in more than 25% of patients referred to them with tic disorders (Supplementary Figure 6). The issue of functional tics overlaid on a primary tic disorder was also reported by 21% of respondents in more than 25% of their patients (Supplementary Figure 7). The most influential clinical factors raising the suspicion towards the diagnosis of a functional tic disorder included movement disorder semiology, the presence of additional functional neurological signs, sudden onset, and the documentation of psychological stressors (see ***Figure 1*** for details on all the related questions).

### Tic pathophysiology

**Figure 2:**
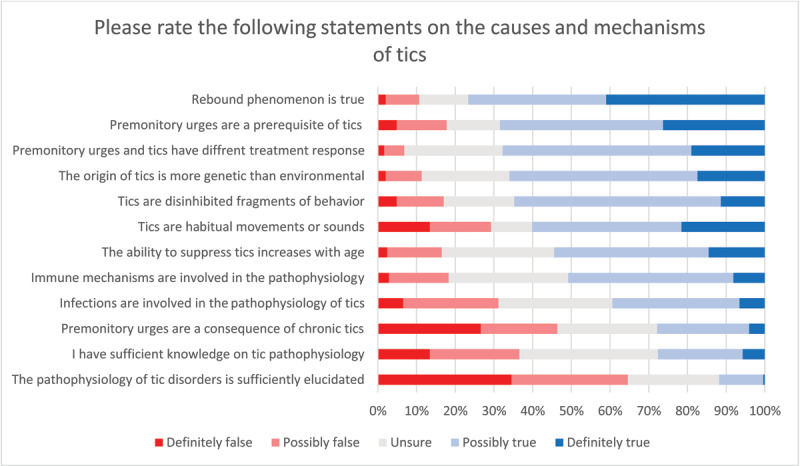
Perceptions on tic pathophysiology. Total number of respondents: n = 248.

Approximately 60% of survey respondents felt confident (when answers from the *possibly true* and *definitely true* categories were merged) that tics are habitual movements or sounds. A similar frequency of respondents supported the view that tics are disinhibited fragments of movements. With regard to premonitory urges, about 68% of respondents viewed them as a fundamental pathophysiological prerequisite of tics, contrasted with only 28% who considered that premonitory urges were a consequence of the presence of chronic tics. Interestingly, 68% of respondents saw a distinction between premonitory urges and tics regarding their response to treatment. Most (65%) respondents supported the view that the origin of tics is more genetic than environmental, nearly 51% reported that there might be an immunological basis for their origin, and 39% considered a role of infections in tic pathophysiology. Importantly, more than 65% of respondents did not feel confident that the pathophysiology of tics is sufficiently elucidated, and only 27% were confident about their knowledge on the topic (see ***Figure 2*** for all related questions).

### Treatment and Prognosis

**Figure 3:**
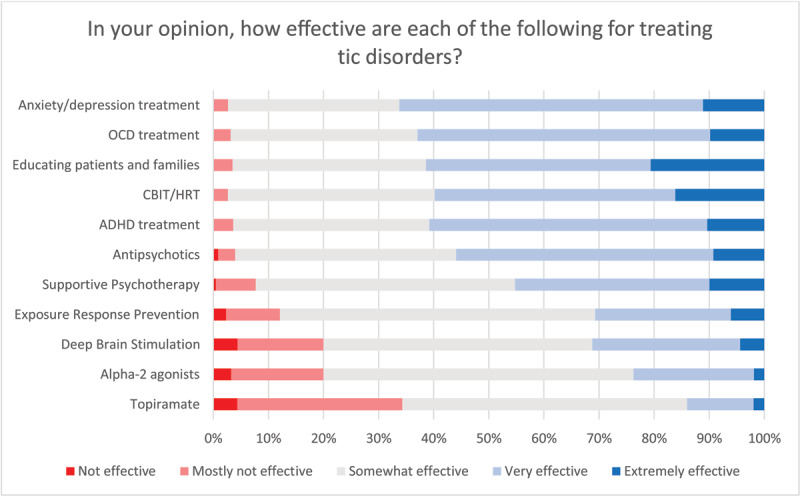
Opinions on treatment efficacy of different interventions. Total number of respondents: n = 231.

Of all survey respondents seeing patients with tic disorders, 88% reported being directly involved in their treatment. Seventy-eight percent reported being aware of published treatment guidelines, 93% of whom also reported using them in their practice. The two most commonly used treatment guidelines are those from the American Academy of Neurology (73%) and the European Society for the Treatment of Tourette’s Syndrome (55%) (Supplementary Figure 8). Regarding preferred treatments for tics, 69% of respondents positioned behavioral therapies as first-line treatment, even though only 54% reported having access to trained therapists. Among behavioral therapies, more clinicians rated comprehensive behavioral intervention for tics (CBIT) very or extremely effective and more beneficial than exposure response prevention (ERP). Interestingly, supportive psychotherapy (the control condition in many clinical trials of behavioral therapies) was also considered by clinicians to be very or extremely effective and again more successful than ERP (see ***Figure 3***).

Of the medication classes used to treat tics, antipsychotics were more commonly rated as ‘very or extremely effective’ than alpha agonists or topiramate. Of all medications, risperidone was the most selected among the top five preferred agents. ***Table 2*** provides a complete list of the top 5 preferred agents for adult and pediatric/adolescent age groups. However, in terms of ranking for most favored medication among all selected substances, aripiprazole received the highest number of first-place votes by most clinicians, followed by clonidine (Supplementary Figure 9). For children, clonidine was the preferred medication for most clinicians, followed by aripiprazole (Supplementary Figure 10). For adults, the most commonly used medications differed between the Americas and Europe. In the Americas, the most commonly used medications were clonidine, aripiprazole, risperidone, tetrabenazine, and topiramate, while in Europe they were aripiprazole, risperidone, quetiapine, botulinum toxin, and tetrabenazine (see Supplementary Tables 3 and 4 for top choices and response rates for adults and children/adolescents, respectively).

**Table 2:** Top 5 selected medications used to treat tics in adults and children/adolescents.


ADULTS	N*	CHILDREN/ADOLESCENTS (N)	N*

Risperidone	129	Risperidone	121

Aripiprazole	126	Aripiprazole	114

Clonidine	105	Clonidine	110

Tetrabenazine	100	Topiramate	76

Haloperidol	88	Haloperidol	75


* = Total number of responses for adults = 220, for children/adolescents = 197.

Educating patients and families and treating neuropsychiatric comorbidities, such as ADHD, OCD, anxiety, and depression, were the most common interventions rated as very or extremely effective for treating tic disorders. Deep brain stimulation was considered to be very or extremely effective by nearly 30% of the respondents, with approximately four percent not considering it as effective at all (see ***Figure 3*** for all relevant queries).

The most frequently selected factors hindering treatment success of patients with tic disorders were financial coverage of treatments, access to referral sources, and additional neuropsychiatric symptoms (Supplementary Figure 11). With regard to overall prognosis, the top three factors suggested to exert an influence were the presence and number of neuropsychiatric comorbidities (77%), tic severity at the time of presentation (66%), and the response to treatments (56%). Also, 22% of respondents suggested that the presence and severity of premonitory urges and the ability to exert volitional inhibitory tic control (21%) are relevant factors driving prognosis. Another 9% reported female sex to influence overall tic prognosis (Supplementary Figure 12).

## Discussion

This survey provides key insights into the perception and practices of movement disorders clinicians worldwide related to tic disorders and Tourette syndrome. Indeed, although the overall number of survey respondents was comparably low, there was a good representation of movement disorders clinicians with different levels of experience and 72 different countries. Of note, the majority of survey respondents (64%) were below the age of 45. Beyond general factors, such as motivation to participate in an online survey, differences in exposure to the entire range of movement disorders as part of structured fellowship programs could potentially explain this finding. Somewhat surprisingly, only about half of survey respondents reported seeing more than ten patients with tic disorders a year. This might reflect that, despite the common occurrence of tics in the general population, movement disorders clinicians are probably the primary specialist for only the minority of patients with tic disorders. Indeed, psychiatrists, pediatricians, and pediatric neurologists are often the first to see patients with tic disorders. Movement disorders experts are typically consulted when there is ambiguity on the phenomenology of the movement disorder (i.e. whether a motor behavior is a tic or not), or when tics are severe, refractory to first-line therapies, or if botulinum toxin injections are being considered. It may also be the case that a few experts who subspecialize in tics manage a disproportionate amount of tic patients.

Our survey shows that movement disorders clinicians assess the presence and severity of tics and comorbid neuropsychiatric conditions primarily through narrative interviews rather than structured interviews or standardized screening tools. Several rating scales are available to measure tic severity, with the Yale Global Tic Severity Scale (YGTSS) being the most extensively used worldwide. Rating scales are valuable tools to evaluate the repertoire of tics and treatment response in the clinical setting [10]. North American and European guidelines consider their application useful but acknowledge that practice variation is acceptable in the context of good clinical practice. Time constraints in routine clinical practice may also influence the application of standardized scales. Nevertheless, the high frequency of reported screening for ADHD, OCD, anxiety, and depression is reassuring and in line with existing guidelines.

When endeavoring to rule out secondary etiologies, more than 90% of respondents reported using neuroimaging to rule out contributory causes of tics in their patients, and 66% also ordered laboratory tests, for example, to search for acanthocytes or assess serum copper or ceruloplasmin. Of note, in cases with a typical clinical history and presentation for primary tic disorders and the absence of severe neurodevelopmental issues or additional neurological or other systemic signs, the range of differential diagnoses is minimal. Therefore brain MRI or laboratory tests, including copper studies, represent very low-yield investigations. Other investigations that appear to be used disproportionately for their clinical need include neurophysiology, as for example EEG, which in rare cases may be helpful particularly where overlapping phenomena of different etiologies are suspected [11]. Finally, about one-third of respondents measure Group A Streptococcus antibody (GAS) titers, likely to screen for PANS/PANDAS. Given the relative rarity of acute onset tics associated with other clinical features that define the PANS spectrum (e.g., obsessive-compulsive symptoms, anxiety, behavioral regression, eating behavior abnormalities), the observed frequency of use of this type of screening may also appear unjustified. Of note, a recent multicenter study exploring the relationship between tic exacerbations and GAS titers in 715 children with primary tic disorders over 16 months did not find evidence for any association between the two [12]. The reported frequency of ancillary investigations to screen for secondary causes of tics could be related to movement disorders clinicians evaluating more complex and atypical cases of tic disorders than other specialists, including adult cases or reflect different practice habits within movement disorders compared to other specialists clinics. At the same time, it may have also resulted in part from how the question was interpreted and could be clarified by determining how often clinicians suspect secondary causes. There is still probably an insufficient understanding of the phenomenological and syndromic differences between primary and secondary tics, which large observational clinical cohorts could address.

Functional tics were reported to be the most common differential diagnosis to primary tics. This highlights that the occurrence of tics due to functional neurological disorder is not a rare observation. More recently, a global surge of cases with explosive onset tic-like behaviors in adolescents, many of which are due to a functional neurological disorder, may explain the higher frequency of functional tics reported in this survey [13]. Of note, the diagnostic distinction between primary and functional tics may be notoriously difficult, and although good clinical classifiers have been suggested [14,15,16], there may be significant overlap in their presentation [17]. Importantly, in some cases, both primary and functional tics may co-occur, and indeed about one-fifth of respondents documented functional overlay in more than 25% of their patients referred with tics. In the absence of reliable tools to distinguish akin clinical phenomena with different etiologies, these results should be interpreted cautiously and highlight the need for further research in this domain.

Beyond diagnostic assessment and etiologic labeling, survey responses about pathophysiology revealed that both habit formation [18] and disinhibition [19] theories have significant traction. Recently, a framework has been proposed combining both theories to explain the presence of tic behaviors [20]. Of note, we detected uncertainty concerning knowledge or understanding of the pathophysiology of tic disorders, with only 27% of respondents feeling confident of their knowledge on the topic. This low confidence may be due to limited global dissemination of information in tic disorders, even among movement disorders specialists, and the underlying need for more research on this topic. Noteworthy is also that about 40% of respondents felt that infections might be intrinsically involved in the pathophysiology of some tics (answering “possibly true” or “definitely true”). The association between infections and primary tic disorders and OCD is supported, albeit not unanimously, by epidemiologic studies based on healthcare registries [21,22,23]. At the same time, this association is not observed in prospective longitudinal studies of patients with tic disorders [12,24]. The observed frequency of survey responders supporting a pathophysiologic role of infections in tics may stem from this controversial evidence. It would be advantageous to explore also how much the perception of a clinical link between primary tic disorders and PANS/PANDAS influences this judgment.

Among the range of different treatment options, survey participants rated psychoeducation as the most important intervention to treat tics, which aligns with the existing guidelines recommending referring people with TS to resources for psychoeducation for teachers and peers [6]. It is important to note that the management of comorbid conditions such as OCD, anxiety, and depression, and the application of behavioral treatment protocols, specifically CBIT, were the most highly rated interventions to treat tics after psychoeducation. Although CBIT is indeed the first-line recommendation of several treatment guidelines, the treatment of comorbidities before tics to reduce the latter’s presence might appear somewhat counterintuitive. However, it is essential to note that clinically relevant anxiety, OCD, and depression symptoms might worsen tic severity and substantially deteriorate patients’ quality of life irrespective of tic severity. Given that the interplay between tic severity, neuropsychiatric comorbidities, and quality of life is complex [25,26,27], an individually tailored therapeutic approach based on a hierarchical listing of all clinically relevant problems is required. Somewhat not surprising in this regard, tic severity, the number of neuropsychiatric comorbidities, and the response of tics to treatment interventions were rated as the most relevant prognostic factors to influence the persistence of tics in adulthood.

Respondents to this survey reported using clinical practice guidelines on the assessment and management of tics, and their choices for the top five medications used in children and adults are mostly in keeping with current evidence-based recommendations. Clonidine was ranked the number one favored medication in children, likely since current evidence for the use of clonidine for tics is stronger in children with tics and comorbid ADHD [6] and its more benign adverse effect profile compared to antipsychotics. Further, some published treatment algorithms highlight alpha-agonists among first-line pharmacologic options ([5,6]). Risperidone and aripiprazole were indicated as the two most favored medications in adults. This finding reflects the efficacy of these two antipsychotics in reducing tic severity, demonstrated by systematic reviews [6]. Haloperidol is similarly effective but appears to be less favored due to its poor tolerability. Tetrabenazine was among the top five medications prescribed to adults. To date, there are no published randomized controlled trials of tetrabenazine for the treatment of tics, and recently completed randomized controlled trials of deutetrabenazine and valbenazine failed to demonstrate a difference with placebo for the primary outcome [28]. When contrasting top choices for medication by region, quetiapine appeared in the top five for European practitioners when treating adults. Evidence to support the use of quetiapine for tics is limited to a few small open-label studies [29,30]. Quetiapine has limited D2 antagonism at tolerable doses (less than 300 mg), which likely limits its therapeutic potential for tics. It is important to note that respondents rated most favored agents used, not necessarily most potent in efficacy. Choice of agent likely reflects both goal of tic-reduction and also adverse effect avoidance.

We acknowledge some relevant limitations of our study. As shared in extensive survey studies using a similar design to ours, we obtained a low response rate among members of the MDS, which might have skewed our results towards a self-selected sample of academic (or research-oriented) clinicians. The academic profile of the majority of respondents might explain in part the relatively low number of patients with tic disorders seen in one year compared to other categories of frontline clinicians. Also, while the majority of patients seeking medical attention for tics are children, the majority of survey respondents (43%) reported exclusively seeing adult patients, which also reflects the overall structure of the MDS membership. Therefore, our results should be interpreted keeping in mind the surveyed population, without generalizing to the whole community of clinicians evaluating or managing patients people with tics. Some geographic areas, e.g., Africa and Oceania, are under-represented in our survey (7% and 1% of all survey responses, respectively), which on the one hand could reflect the geographic distribution of the MDS membership, but may also indicate practice and cultural differences in the role of movement disorders clinicians in the healthcare provision for persons with tics. This deserves to be explored further in studies explicitly conducted in these under-represented areas. Finally, the relatively large number of survey questions might have contributed to differences in response rate across our survey questions and sections. This should be considered in the design of future survey-based investigations within the MDS and societies with a similar academic and professional membership profile.

In summary, we here provide the first global survey to capture perceptions and practices of movement disorders clinicians related to tic disorders and Tourette syndrome. The survey highlights that despite their high occurrence in the general population, tics remain a relatively uncommon movement disorder presentation for most movement disorders clinicians. Together with the complexity and diversity of clinical symptoms, this fact conveys the need for further standardization of assessment practices, including how to approach different possible etiologies. More work should also be invested in research and knowledge dissemination on pathophysiologic and etiological aspects of tics, as this constitutes the basis for informed clinical practice, including selecting evidence-based and individually-tailored treatments across the globe.

## Additional Files

The additional files for this article can be found as follows:

10.5334/tohm.656.s1 Supplementary material 1. The survey.

10.5334/tohm.656.s2 Supplementary material 2. Additional Figures and Tables.
