# Supplementary material for: Clinical Practice Patterns in Tic Disorders Among Movement Disorder Society Members
Source: Tremor Other Hyperkinet Mov (N Y). 2021 Oct 28;11:43. doi: 10.5334/tohm.656 (PMC8555622; doi:10.5334/tohm.656)
Supplement: Supplementary material 1. — The survey. [file tohm-11-1-656-s1.pdf]

Supplementary material 1. The survey

I. DEMOGRAPHIC INFORMATION

1. Age (years)
[ ] 25-35 [ ] 36-45 [ ] 46-55 [ ] 56-65 [ ] >66

2. Years in practice? (Post-residency/fellowship)
[ ] < 5 years [ ] 6-10 years [ ] 11-15 years [ ] 16-20 years [ ] >21 years

3. Which best describes your practice?
[ ] Academic clinician working in a hospital
[ ] Non-academic clinician working in a hospital
[ ] Academic clinician working in a community service
[ ] Non-academic clinician working in a community service

4. In what country do you currently practice?

5. What is the number of patients with <u>tic disorders</u> that you see on average?
[ ] No patients [ ] 1-10 per year [ ] 1-10 per month [ ] >10 per month

If No Patients survey ends.

6. What is the percentage of patients with tic disorders of your total volume of patients with movement disorders that you saw on average during the past year? (please enter number in %)

II. SURVEY ON TICS AND TIC DISORDERS

1. **What is the age range of the patients with tic disorders that you see in your usual clinical practice?**
   - Exclusively adults
   - Mostly adults
   - Adults and adolescents (12-18 years of age)
   - Adults, adolescents and children (<12 years of age)
   - Mostly adolescents and children
   - Exclusively adolescents and children

2. **Beyond primary tic disorders, which conditions manifesting with secondary tics do you see in your practice? Please indicate all those that apply.**

   - Tics in neurodegeneration (e.g. Huntington's chorea, Chorea-Acanthocytosis)
   - Tics in neurometabolic disorders
   - Tics in autism spectrum disorders
   - Tics in other neurogenetic conditions
   - Tics as a result of structural brain injury
   - Tics induced by medication
   - Tics (or tic-like movements) in functional neurological disorders

3. **Do you systematically document the presence of the following additional tic-related behaviors? Please indicate all that apply.**

   - Echophenomena (e.g. echolalia, echopraxia)
   - Coprophenomena (e.g. coprolalia, copropraxia)
   - Paliphenomena (e.g. palilalia, palipraxia)
   - Non-obscene inappropriate behaviors (NOSIs)
   - Self-injurious behaviours (SIBs)

4. **How frequently do you use standardized scales for the assessment of tic severity in your clinical practice?**

   - In more than 75% of patients
   - Between 50-75% of patients
   - Between 25-50% of patients
   - In less than 25% of patients
   - I never use standardized scales (If selected Question No 5 should be skipped)

5. **Which standardized scales for the assessment of tic severity do you use in your clinical practice? Please indicate all which apply**

   - Yale Global Tic Severity Scale
   - Modified Rush Video Protocol
   - Tourette Syndrome – Global Clinical Impression
   - MOVES
   - Others (please name)

6. **Do you screen for the presence of neuropsychiatric comorbidities (e.g. obsessive-compulsive disorder: OCD, attention-deficite hyperactivity disorder: ADHD, anxiety disorder, depression) in patients with tic disorders in your clinical practice?**

   - Yes
   - No (If selected Questions 7-10 should be skipped)

7. **How do you assess OCD in your clinical practice?**

   - By clinical interview
   - Use of structured interviews
   - Use of rating scales
   - I do not assess/not applicable

   If CLICK for Rating Scales:

   Which rating scales do you use?

   - Yale-Brown Obsessive Compulsive Scale (Y-BOCS)
   - Children's Yale-Brown Obsessive Compulsive Scale (CY-BOCS)
   - Others (please name)

8. **How do you assess ADHD in your clinical practice?**

   - By clinical interview

- Use of structured interviews
- Use of rating scales
- I do not assess/not applicable

If CLICK for Rating Scales:

Which rating scales do you use?

- ADHD Self Report Scale (ASRS)
- Conners' Adult ADHD Rating Scale
- Conners' ADHD Rating Scale
- SNAP-IV
- Vanderbilt ADHD Diagnostic Rating Scale
- Others (please name)

9. How do you assess anxiety disorder in your clinical practice?

- By clinical interview
- Use of structured interviews
- Use of rating scales
- I do not assess/not applicable

If CLICK for Rating Scales:

Which rating scales do you use?

- Hospital Anxiety and Depression Scale (HADS)
- Hamilton Anxiety Rating Scale (HAM-A)
- Beck Anxiety Inventory (BAI)
- Screen for Child Anxiety Related Disorders (SCARED)
- Multidimensional Anxiety Scale for Children
- Others (please name0

10. How do you assess depression in your clinical practice?

- By clinical interview
- Use of structured interviews
- Use of rating scales
- I do not assess/not applicable

If CLICK for Rating Scales:

Which rating scales do you use?
- Beck Depression Inventory (BDI)
- Hamilton Depression Rating Scale (HAM-D)
- Children's Depression Inventory (CDI)
- Others (please name0

11. Do you routinely use any additional investigations to rule out secondary causes of tic disorders in your patients? Please indicate all which apply.

- Neuroimaging (e.g. MRI, CT)

- Neurophysiology, including surface polymyography, EEG, EEG-EMG coupling
- Streptococcal serology to screen for PANS/PANDAS
- Cunningham panel
- Other blood investigations (e.g. search for acanthocytes, serum copper levels/ceruloplasmin)
- Urinary copper excretion

12. Of all the patients referred to you for tics or tic-like behaviors, what is the percentage in whom you diagnose a functional tic-like disorder?

- >50%
- 25-50%
- 10-25%
- 5-10%
- <5%

13. Of all the patients with tic disorders you follow, what is the percentage of those who have co-existing tics and functional tic-like behaviours?

- >50%
- 25-50%
- 10-25%
- 5-10%
- <5%

14. How influential are each of the following in supporting a diagnosis of <u>a functional tic-like disorder</u>

	Not influential at all	Mostly not influential	Somewhat influential	Very influential	Extremely influential	Not sure
Age at onset (i.e., >18 years)	O	O	O	O	O	O
Sudden onset	O	O	O	O	O	O
Semiology (i.e. predominant complex tics, lack of rostrocaudal tic gradient)	O	O	O	O	O	O
Presence of coprophenomena	O	O	O	O	O	O
Inability to voluntarily suppress tics	O	O	O	O	O	O
Lack of premonitory urges	O	O	O	O	O	O
Absence of Echo- or Paliphenomena	O	O	O	O	O	O
Female Sex	O	O	O	O	O	O
Presence of other functional neurological	O	O	O	O	O	O

signs (e.g. functional weakness, functional gait disorder etc)						
Presence of psychological stressors (e.g. traumatic events)	O	O	O	O	O	O
Normal findings on ancillary examinations (e.g. MRI, laboratory investigations, EEG)	O	O	O	O	O	O
Presence of comorbidities such as depression or anxiety disorders	O	O	O	O	O	O

15. Which do you consider as predictive factors for persistence of tics in adulthood? Please indicate three or less.

- Tic severity at the time of presentation
- Number and severity of neuropsychiatric comorbidities
- Response to treatments
- Presence and severity of premonitory urges
- Capacity to voluntarily suppress tics
- Female Sex
- Other (please indicate)

16. Please rate the following statements on the causes and mechanisms of tics

	Definitely false	Possibly false	Unsure	Possibly True	Definitely true
Tics are habitual movements or sounds	O	O	O	O	O
Tics are disinhibited fragments of behavior	O	O	O	O	O
Premonitory urges are a fundamental prerequisite of tics in adolescents and adults	O	O	O	O	O

Premonitory urges are a consequence of having had chronic tics	O	O	O	O	O
Premonitory urges and tics can respond differently to active treatments	O	O	O	O	O
The ability to suppress tics increases with age	O	O	O	O	O
In most patients, after a period of tic suppression, tics restart more intensely (rebound phenomenon)	O	O	O	O	O
The origin of tics is more genetic than environmental	O	O	O	O	O
Infections are involved in the pathophysiology of tics	O	O	O	O	O
Immune mechanisms are involved in the pathophysiology of tics	O	O	O	O	O
I have sufficient knowledge on tic pathophysiology	O	O	O	O	O
The					

pathophysiology of tic disorders is sufficiently elucidated	O	O	O	O	O

17. Do you directly provide treatment for patients with tic disorders that you assess or diagnose (i.e. prescribe medication, organize behavioral treatment)?

- Yes
- No

18. Are you aware of the presence of treatment guidelines for tic disorders?

- Yes
- No

ONLY IF YES QUESTIONS 19/20 appear

19. Are these guidelines helpful to inform your therapeutic decision?

- Yes
- No

20. Which guidelines do you find informative for your therapeutic decision-making?

- Guidelines of the European Society for the Study of Tourette Syndrome (ESSTS)
- American Academy of Neurology guidelines for the treatment of tic disorders
- Canadian Guidelines for the treatment of tic disorders
- Others (please name)

21. Do you favor behavioral therapy (e.g. Comprehensive Behavioral Intervention for the Treatment of Tics/Habit Reversal, Exposure Response Prevention) as first line treatment?

- Yes
- No
- Only in a subgroup of patients (please indicate the main characteristics of this subgroup)

22. Do you have local access to behavioral therapists for these treatments?

- Yes
- No

23. Please list the top five pharmacological agents you use to treat tics in adults starting from the most favored one.

"Pharmacological Agents (others may apply)"

Aripiprazole,
Atomoxetine,
Botulinum toxin,
Cannabinoids,
Clonidine,
Guanfacine,
Haloperidol,
Nicotin patches,
Paliperidone,
Pimozide,
Quetiapine,
Risperidone,
(Ami)Sulpiride,
Tetrabenazine,
Thioridazine,
Tiapride,
Topiramate,
Ziprasidone,
Others – please name

24. Please list the top five pharmacological agents you use to treat tics in children or adolescents starting from the most favored one.

"Pharmacological Agents (others may apply)"

Aripiprazole,
Atomoxetine,
Botulinum toxin,
Cannabinoids,
Clonidine,
Guanfacine,
Haloperidol,
Nicotin patches,
Paliperidone,
Pimozide,
Quetiapine,
Risperidone,
(Ami)Sulpiride,
Tetrabenazine,
Thioridazine,
Tiapride,
Topiramate,
Ziprasidone,
Others – please name

25. In your opinion, how effective are each of the following for treating tic disorders?

	Not effective at all	Mostly not effective	Somewhat effective	Very effective	Extremely effective
Educating patients and families	O	O	O	O	O

Comprehensive Behavioral Intervention for the Treatment of Tics/Habit Reversal	O	O	O	O	O
Exposure Response Prevention	O	O	O	O	O
Supportive Psychotherapy	O	O	O	O	O
Antipsychotics	O	O	O	O	O
Alpha-2 agonists	O	O	O	O	O
Topiramate	O	O	O	O	O
Pharmacological and/or behavioral treatment of OCD (when present)	O	O	O	O	O
Pharmacological and/or behavioral treatment of ADHD (when present)	O	O	O	O	O
Pharmacological and/or behavioral treatment of anxiety/depression (when present)	O	O	O	O	O
Deep Brain Stimulation	O	O	O	O	O

26. How often does each of the following limit your ability to manage patients with tic disorders?

	Never	Rarely	Sometimes	Often	Always
Individual expertise	O	O	O	O	O
Financial coverage of therapies, including referral services	O	O	O	O	O

Geographic availability of referral services	O	O	O	O	O
Presence of additional neuropsychiatric symptoms (e.g., ADHD, OCD, Depression, Anxiety)	O	O	O	O	O
Patient or parental compliance	O	O	O	O	O

Additional Comments: Are there any other issues that you believe are important in the diagnosis and treatment of tic disorders and Tourette syndrome that have not been addressed?
