# Supplementary material for: Clinical Practice Patterns in Tic Disorders Among Movement Disorder Society Members
Source: Tremor Other Hyperkinet Mov (N Y). 2021 Oct 28;11:43. doi: 10.5334/tohm.656 (PMC8555622; doi:10.5334/tohm.656)
Supplement: Supplementary material 2. — Additional Figures and Tables. [file tohm-11-1-656-s2.pdf]

Supplementary material 2. Additional Figures and Tables

Supplementary Table 1. List of participant countries and number of respondents from each country.

Country	Number of respondents
United States	38
Brazil	24
India	22
Italy	22
Argentina	17
Slovakia	16
Germany	13
Mexico	10
Russia	10
United Kingdom	10
Canada	9
Egypt	8
Nigeria	8
Spain	8
Ukraine	8
Romania	7
Indonesia	6
South Korea	6
Peru	5
Colombia	4
Croatia	4
France	4
Japan	4
Poland	4
Bangladesh	3
Chile	3
China	3
Greece	3
Israel	3
Philippines	3
Serbia	3
Thailand	3
Turkey	3
Australia	2
Bulgaria	2
Cuba	2
Denmark	2
Ethiopia	2
Malaysia	2
Mongolia	2
Switzerland	2
Vietnam	2

Algeria	1
Armenia	1
Austria	1
Belgium	1
Bosnia and Herzegovina	1
Cameroon	1
Democratic Republic of the Congo	1
Equador	1
Ghana	1
Honduras	1
Hungary	1
Iraq	1
Ireland	1
Kuwait	1
Kyrgyzstan	1
Laos	1
Mali	1
Myanmar	1
Netherlands	1
Norway	1
Pakistan	1
Portugal	1
Reunion	1
Saudi Arabia	1
Slovenia	1
South Africa	1
Sri Lanka	1
Taiwan	1
Tanzania	1
United Arab Emirates	1
Uruguay	1
Not provided	7

Supplementary Table 2. Frequency and type of implemented scales for assessing tics and comorbidities in clinical practice. N refers to number of respondents. *only validated scales are listed.

Domain	Scale	N
Tics	Yale Global Tic Severity Scale (YGTSS)	123
	Tourette Syndrome – Global Clinical Impression	70
	Modified Rush Video Protocol	20
	Motor tic, Obsessions and compulsions, Vocal tic Evaluation Survey (MOVES)	5
	Others	5*
	Self report scale	
	Skala für abnormale unwillkürliche Bewegungen (SKAUB)	
	Quality of life (QoL)	
	Shapiro TS Severity Scale	

OCD	Yale-Brown Obsessive-Compulsive Scale (Y-BOCS)	77
	Children's Yale-Brown Obsessive-Compulsive Scale (CY-BOCS)	28
	Others	16*
	Dimensional Obsessive- Compulsive Scale	
	Florida Obsessive-Compulsive Inventory (FOCI)	
	Padua Inventory	
ADHD	ADHD Self Report Scale (ASRS)	59
	Conners' Adult ADHD Rating Scale	11
	Conners' ADHD Rating Scale	23
	Swanson, Nolan, and Pelham Rating Scale (SNAP-IV)	16
	Vanderbilt ADHD Diagnostic Rating Scale (VADRS)	12
	Others	18*
	Barkley Adult ADHD Rating Scale (BAARS)	
Anxiety	Hospital Anxiety and Depression Scale (HADS)	56
	Hamilton Anxiety Rating Scale (HAM-A)	54
	Beck Anxiety Inventory (BAI)	56
	Screen for Child Anxiety Related Disorders (SCARED)	5
	Multidimensional Anxiety Scale for Children	6
	Others	15*
	Zung Self-Rating Anxiety Scale	
	Generalized Anxiety Disorders (GAD)-7	
	Social Phobia Inventory (SPIN)	
	Spence Children's Anxiety Scale (SCAS)	
Depression	Beck Depression Inventory (BDI)	113
	Hamilton Depression Rating Scale (HAM-D)	76
	Children's Depression Inventory (CDI)	10
	Others	13*
	Patient Health Questionnaire (PHQ)-9	
	Zung Self Rating Depression Scale	
	Quick Inventory of Depressive Symptomatology (QIDS)	

Supplementary Table 3. Ranked five most used medication in comparison between the Americas and Europe in adults.

Top adult medication in the Americas	N*	Top adult medication in Europe	N*
Clonidine	53	Aripiprazole	48
Aripiprazole	51	Risperidone	39
Risperidone	51	Quetiapine	37
Tetrabenazine	47	Botulinum toxin	36
Topiramate	39	Tetrabenazine	33

* = Total number of responses for adults =82, for children/adolescents =80

Supplementary Table 4. Ranked five most used medication in comparison between the Americas and Europe in children (5th place is shared in both the Americas and Europe).

Top child medication in the Americas	N*	Top child medication in	N *

		Europe	
Clonidine	49	Aripiprazole	42
Risperidone	48	Clonidine	34
Aripiprazole	45	Risperidone	34
Topiramate	36	Tiapride	25
Tetrabenazine	31	Topiramate	22
Guanfacine	31	Haloperidol	22

* = Total number of responses for adults =70, for children/adolescents =67

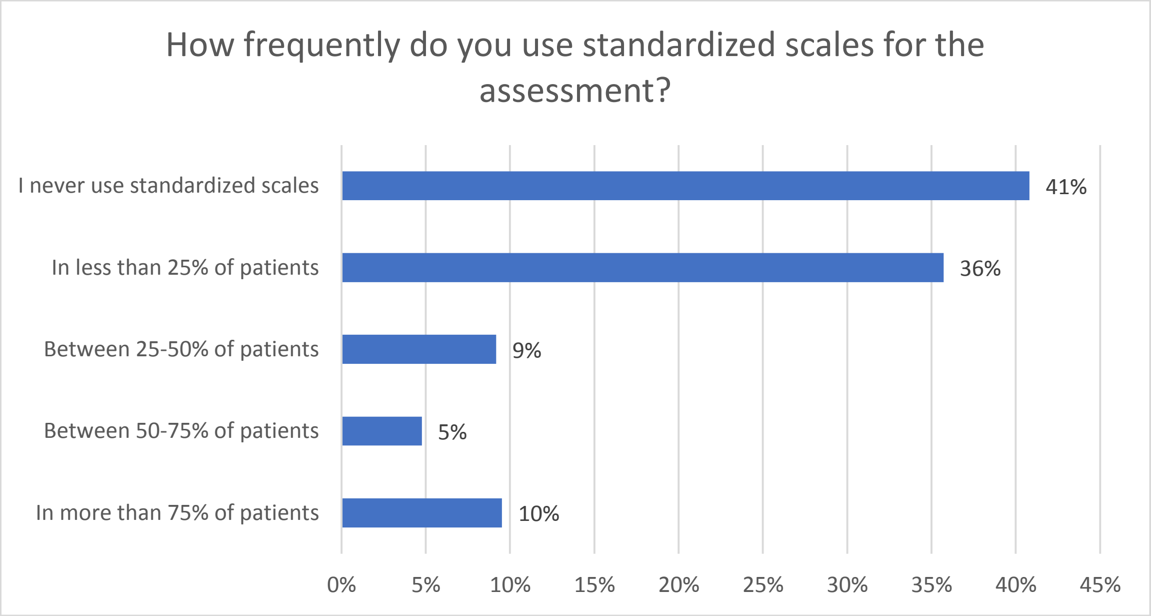

Supplementary Figure 1. Use of standardized tic severity scales in clinical practice. Total number of respondents: n= 294.

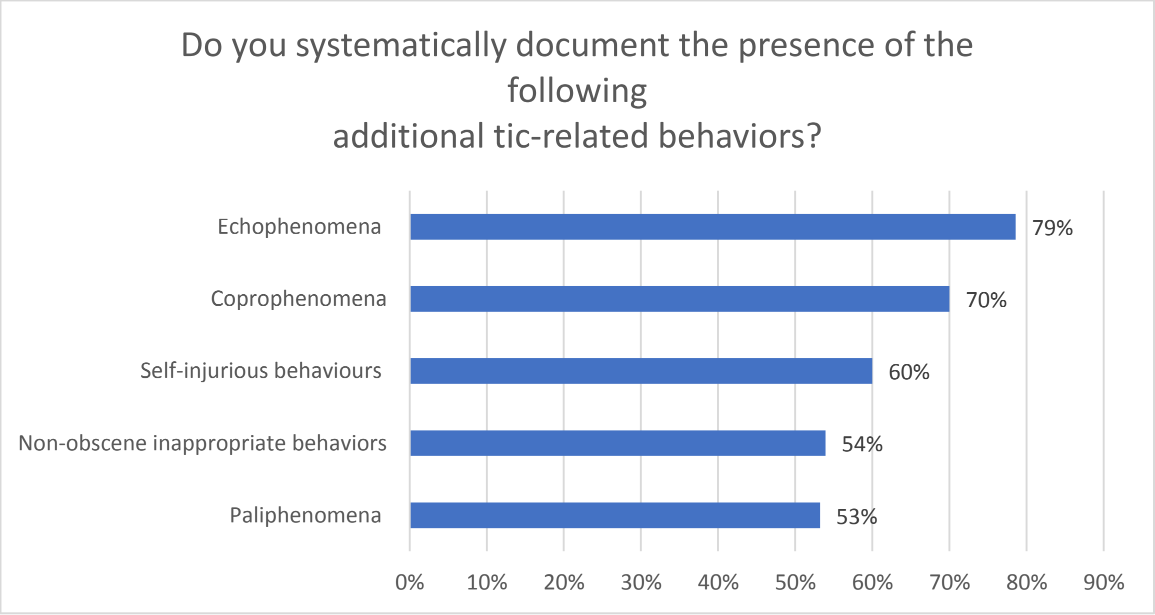

Supplementary Figure 2. Systematical documentation of the presence of additional tic-related behaviors in clinical practice. Multiple choices per participant were possible. Total number of respondents: n=280.

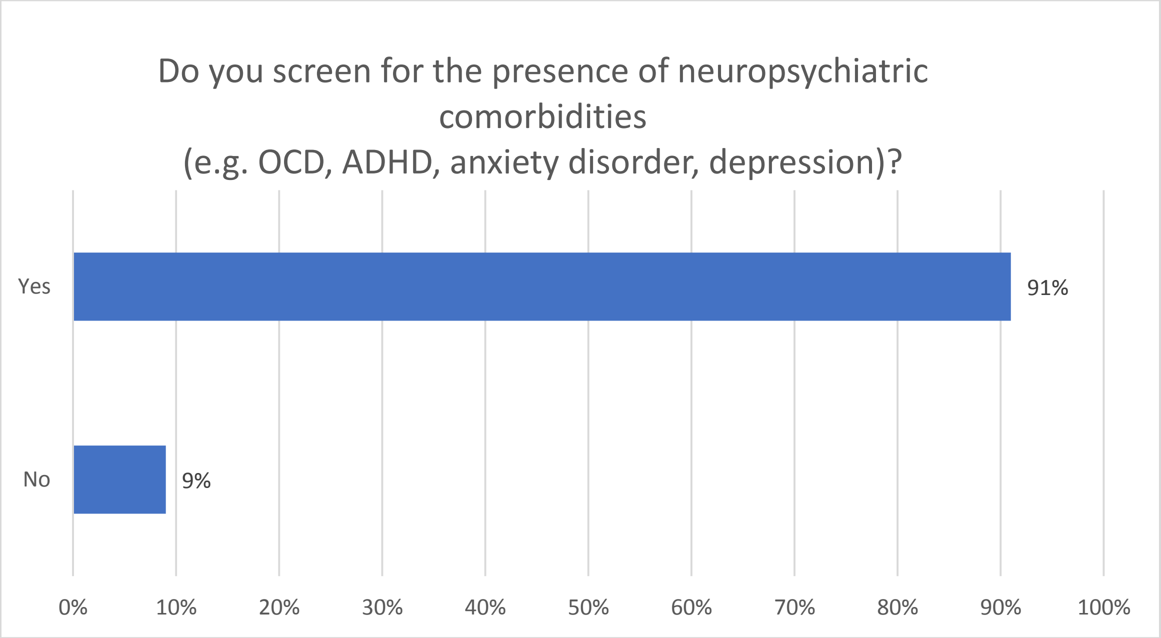

Supplementary Figure 3. Systematical screening of the presence of neuropsychiatric comorbidities in patients with tic disorders. Total number of respondents: n= 289.

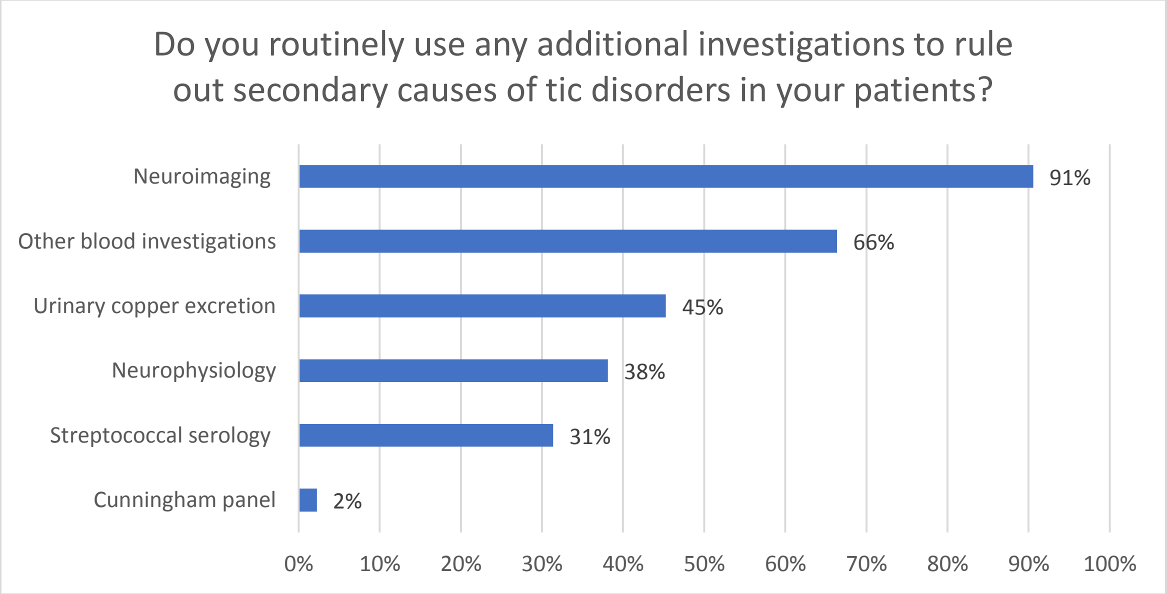

Supplementary Figure 4. Additional investigations for secondary tic disorders. Multiple choices per participant were possible. Total number of respondents: n= 223.

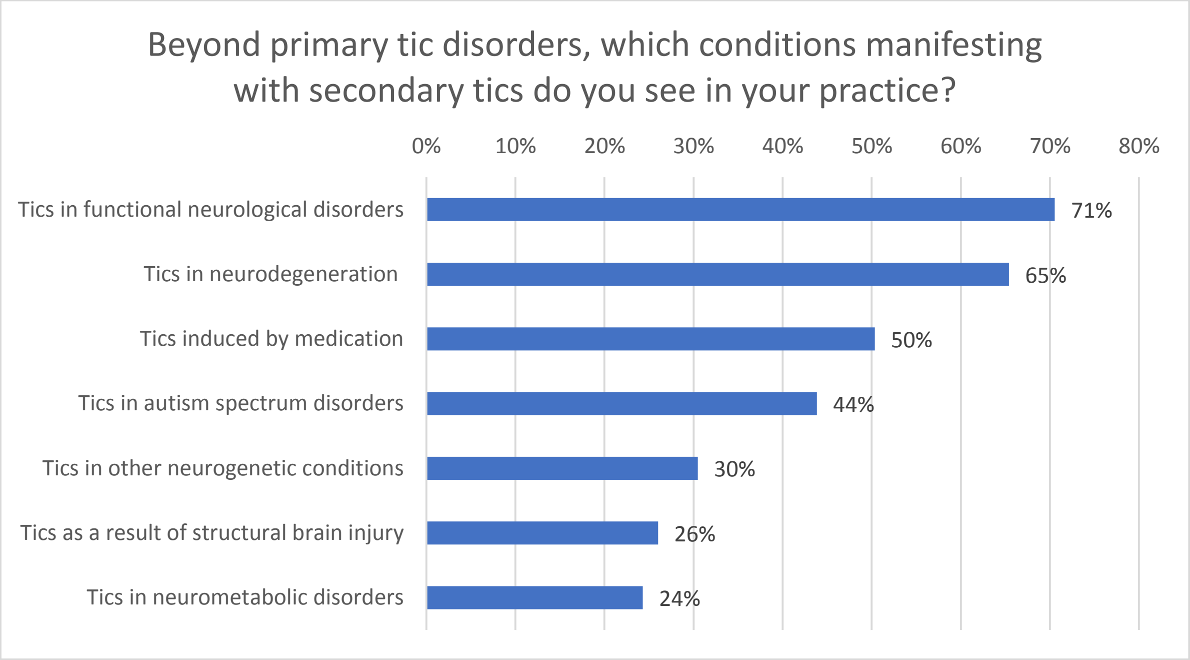

Supplementary Figure 5. List of tic etiologies beyond primary disorders. Multiple choices per participant were possible. Total number of respondents: n= 292.

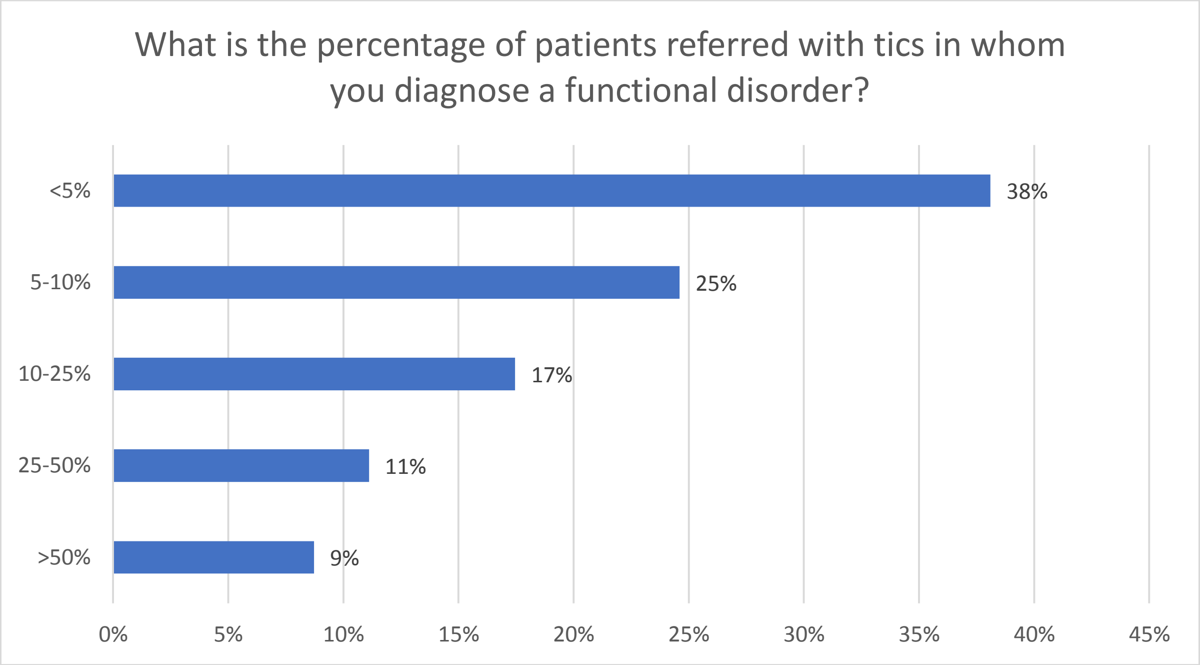

Supplementary Figure 6. Percentage of referred patients presenting with tic-like behaviors subsequently diagnosed with a functional tic-like disorder. Total number of respondents: n= 252.

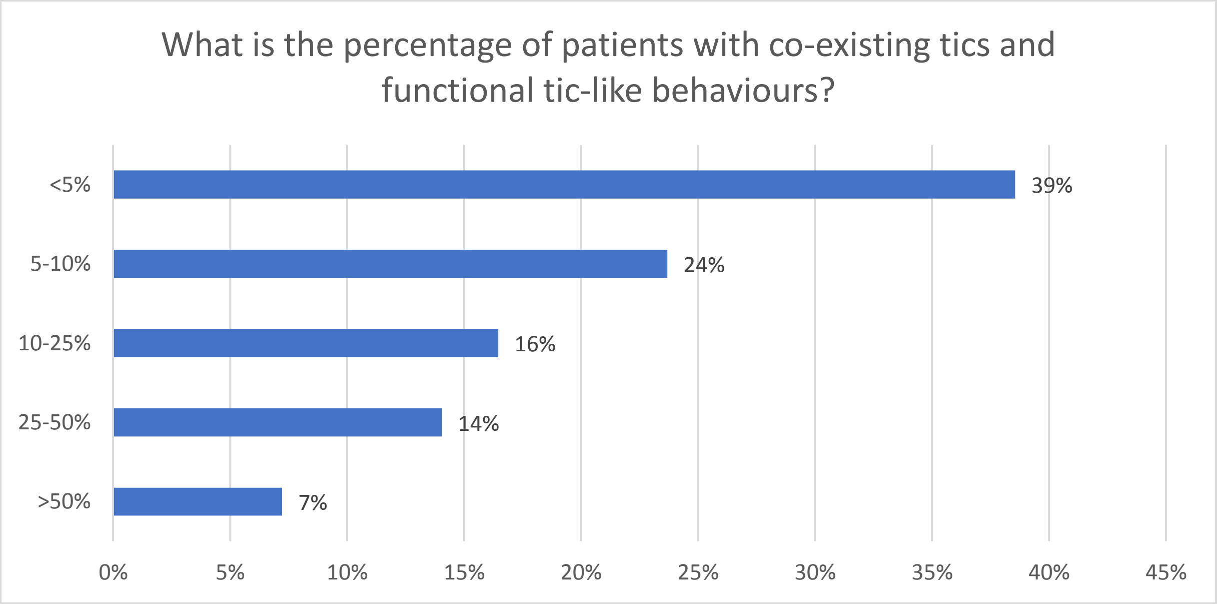

Supplementary Figure 7. Percentage of all patients with tic disorders with functional overlay. Total number of respondents: n= 249.

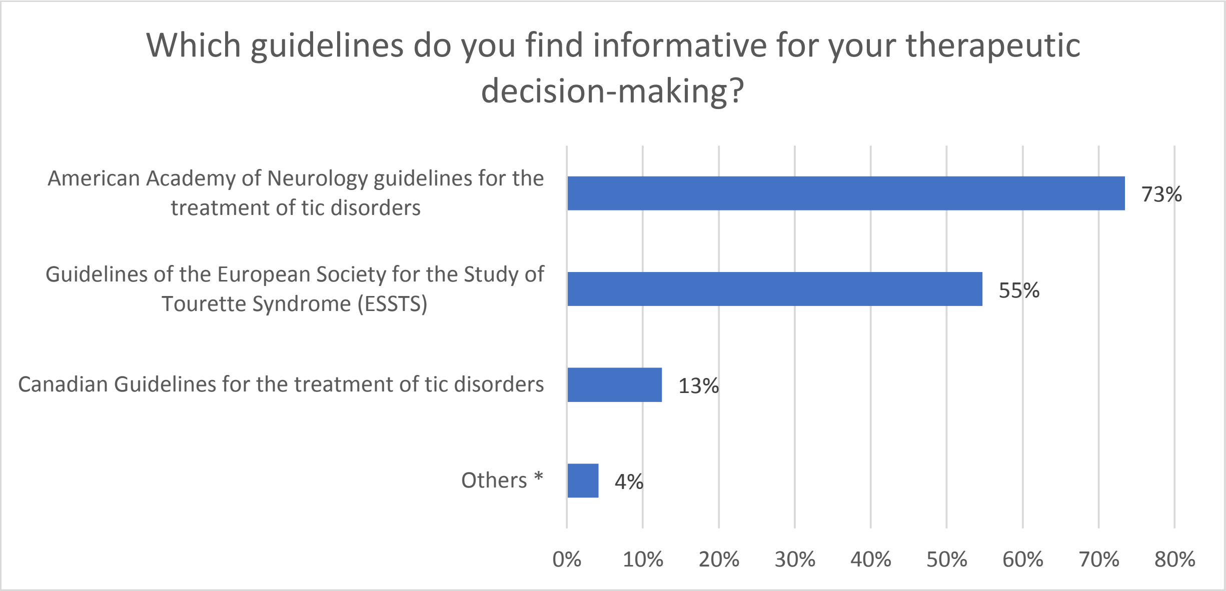

Supplementary Figure 8. Guidelines that were found informative for therapeutic decision-making. Total number of respondents: n= 192.

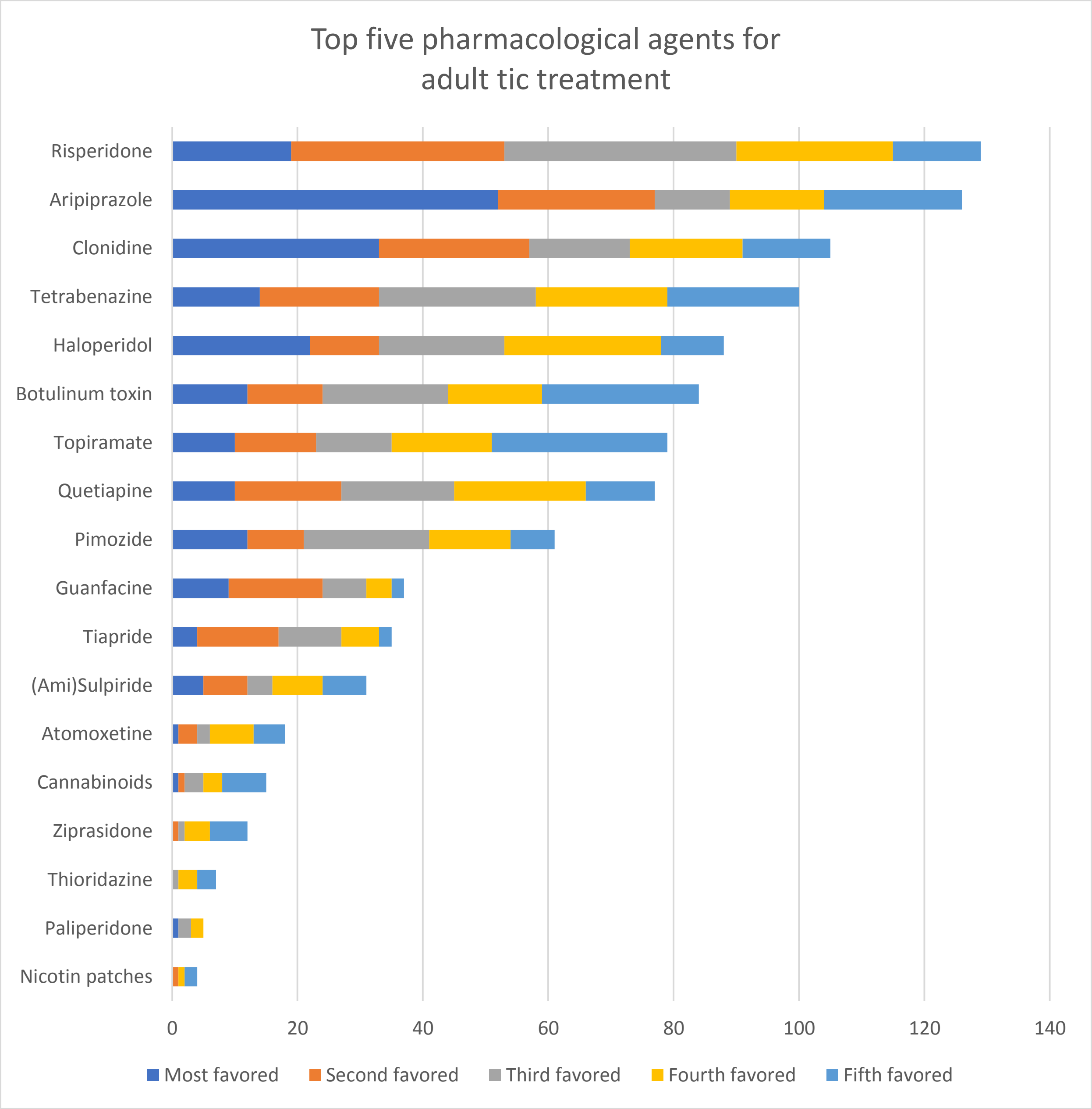

Supplementary Figure 9. Preferred top five pharmacological agents used for treating tics in adults. Total number of respondents: n= 220.

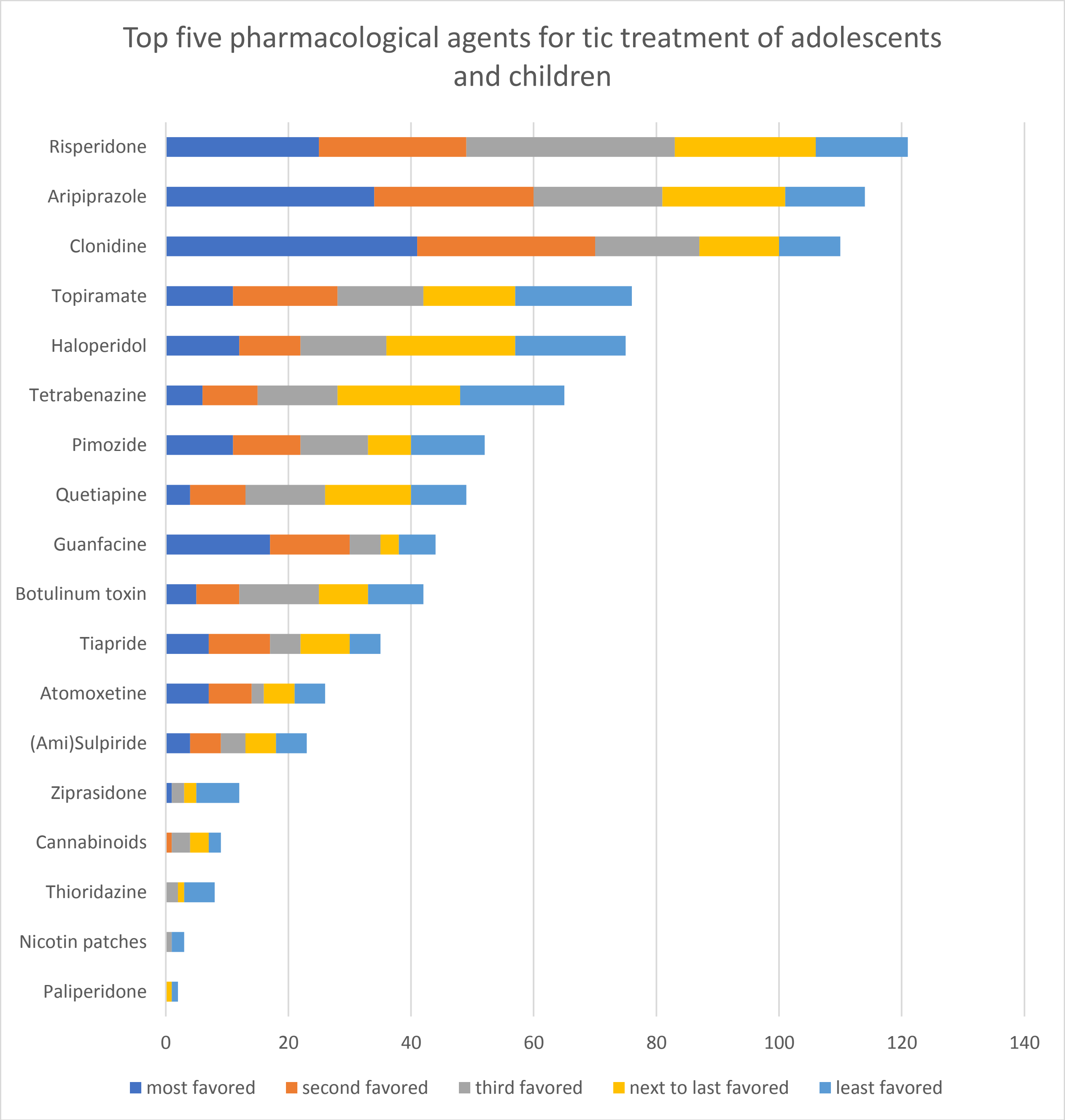

Supplementary Figure 10. The top five pharmacological agents used for treating tics in adolescents and children. Total number of respondents: n= 197.

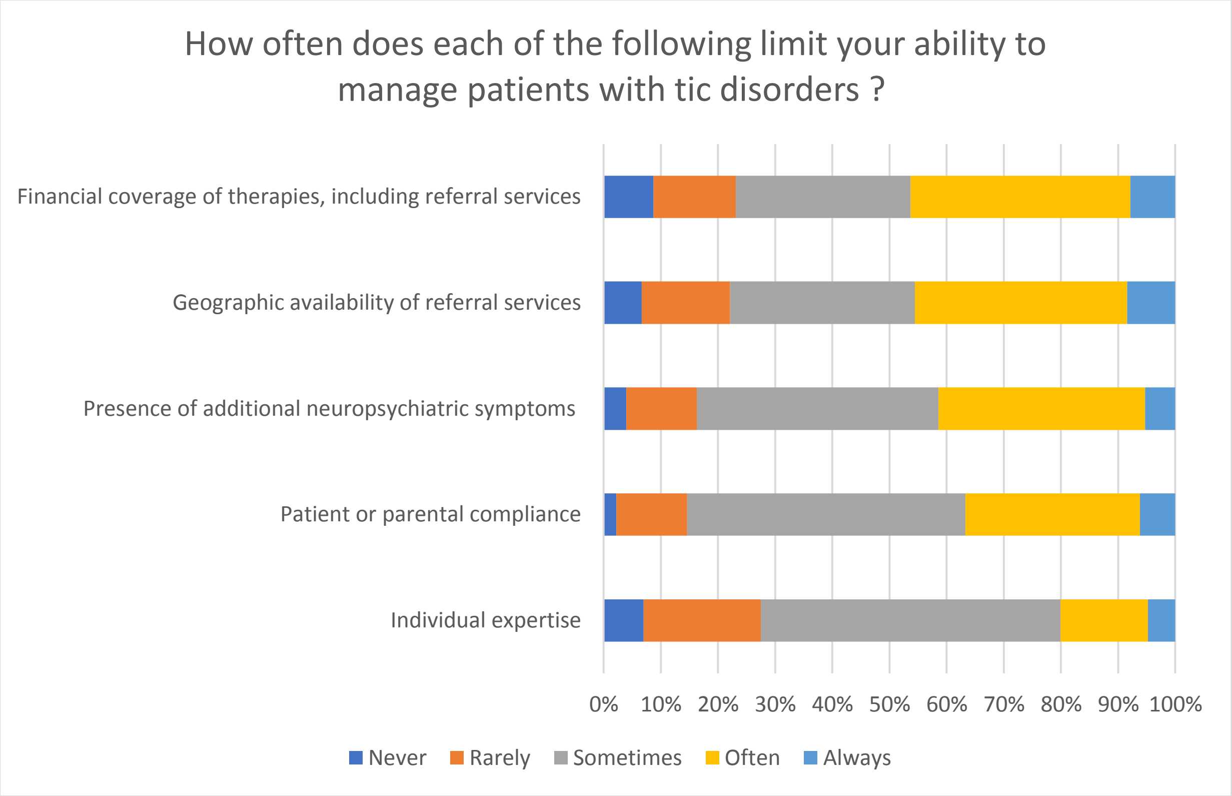

Supplementary Figure 11. Limiting factors to treatment success. Number of respondents: n= 231.

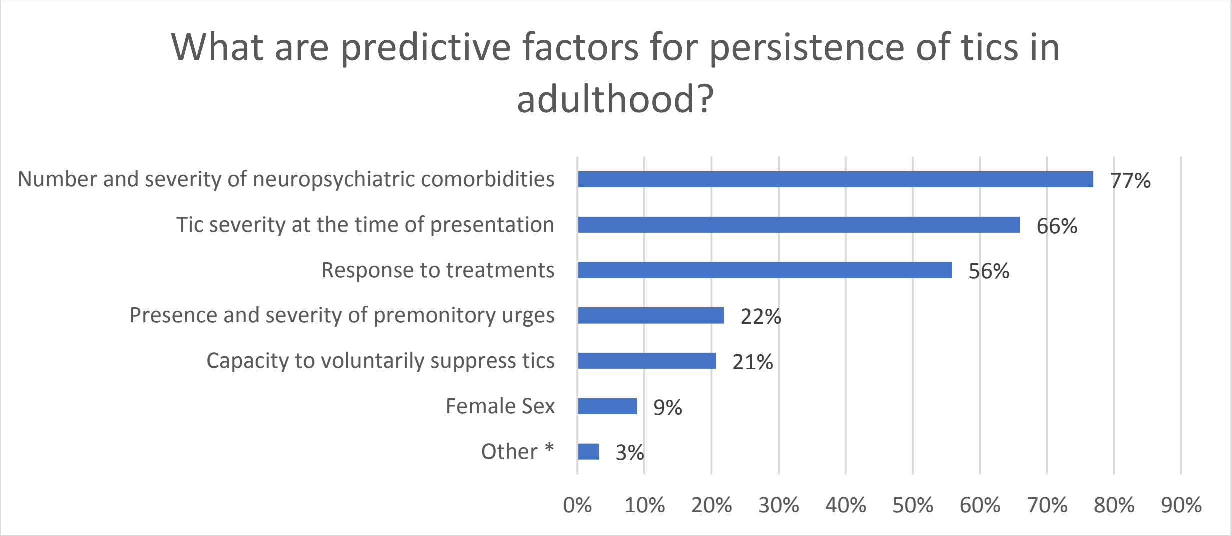

Supplementary Figure 12. Predictive factors suggested to influence prognosis of tic disorders. Multiple choices per participant were possible. Total number of respondents: n= 247.
